# A Possible Perspective about the Compositional Models, Evolution, and Clinical Meaning of Human Enterotypes

**DOI:** 10.3390/microorganisms9112341

**Published:** 2021-11-12

**Authors:** Francesco Di Pierro

**Affiliations:** 1 Digestive Endoscopy & Gastroenterology, Fondazione Poliambulanza, 25124 Brescia, Italy; f.dipierro@vellejaresearch.com; 2 UNICAM, Camerino University, 62032 Camerino, Italy; 3 Scientific Department, Velleja Research, 20124 Milan, Italy

**Keywords:** human gut microbiota, stool consistency, enterotypes, gut microbiota evolution, richness, bacterial load, cardiovascular risk, depression

## Abstract

Among the various parameters obtainable through the analysis of the human gut microbiota, the enterotype is one of the first classifications of the bacterial consortia, which tried to obtain, at the same time, as much information as possible to be applied in clinical medicine. Although some authors observed the existence not of clusters, but only of a real continuous gradient, enterotypes are commonly described according to various models. The first model predicted either clustering into enterotypes 1 and 2 based on two specific dominances, *Bacteroides* and *Prevotella*, respectively, with the *Ruminococcus* dominance blurred within the *Bacteroides* dominance, or it predicted a threedominant condition, in which the *Ruminococcus* driver constituted enterotype 3, separated from enterotype 1. A second model envisaged three possible ways to cluster gut microbiota, respectively centred on two, three, and four dominances. In the first case, enterotypes 1 and 2 coincided with the two original enterotypes, with the dominance of *Bacteroides* and *Prevotella*, respectively. In the second case, the existence of enterotype 3 was evident and whose dominance was not centred on *Ruminococcus* but extended more towards the entire Firmicutes *phylum*. In the third case, the presence of the *phylum* Firmicutes was split into two different enterotypes generating the clusters defined and named as Mixtures 1 and 2. Subsequently, the analysis of the water content (hydration) in the stool allowed the splitting of the *Bacteroides* enterotype into two sub-enterotype, respectively known as B1 and B2. All these models have allowed us to highlight some correlations between a specific enterotype, or cluster, and some characteristics, such as the greater predisposition of the respective hosts towards certain pathologies. These observations, coupled with the attempt to derive the different microbiota on an evolutionary basis, can help to shed new light on this topic and demonstrate the possible utility that the different ways of clustering the gut microbiota can have in a clinical application perspective and in preventive medicine.

## 1. Introduction

In recent years, the results obtained from studies on the human microbiota are so numerous and varied that they can no longer be ignored from a clinical perspective. Unfortunately, the methodological differences adopted by diverse studies are sometimes so considerable that it is difficult to effectively combine their results. However, it is undeniable that, despite these differences in methods, some “facts” could still be considered as, if not consolidated, at least as in the process of being consolidated. For example, a recent meta-analysis has confirmed the strong correlation between a certain structure of the colon fecal microbiota and the possible diagnosis of depression [1]. Elements, such as lower biodiversity, lower representation of the *phylum* Firmicutes, and the lower presence of butyrate-producer *taxa*, such as *Faecalibacterium* and *Coprococcus*, in fact, seem to unite the microbiota of these subjects. If the analysis of the microbiota of a depressed subject were to be superimposed with the same characteristics, a dietary intervention could be planned, which aimed at increasing both biodiversity and the presence of butyrate producers. Both goals could be achieved by modifying the individual’s diet and enriching it with fermented foods [2,3] and dietary fibers [4], and perhaps opting for the use of botanicals, such as curcuminoids [5,6,7] and probiotics, for example *Bifidobacterium adolescentis*, which recently has been experimentally described to counteract a microbiota-driven depressive state [8,9]. To carry out a strategy of this type, however, it is necessary first to highlight, in the gut microbiota, the series of simple and reproducible parameters to which we can entrust our therapeutic choices. This paper, without claiming absolute certainties, tries to propose a narrative report on a gut microbiota parameter, the enterotype, that could be taken into consideration with this precise purpose.

In the last two decades, human gut microbiota studies have generated great attention and, due to the development of metagenomic techniques of bacterial DNA analysis, knowledge of the gut bacterial consortium has recently increased. Gut microbiota is highly diverse and contains trillions of microorganisms mainly belonging to the *phyla* known as Firmicutes, Bacteroidetes, Proteobacteria, Actinobacteria, Verrucomicrobia, Fusobacteria, and Tenericutes [10]. The shaping and diversity of gut microbiome starts at birth, while the modification of their composition depends mainly on various genetic, nutritional, and environmental factors [11]. Alterations in diversity and taxonomic composition of the gut microbiota are thought to be possible drivers of gastrointestinal, metabolic, immunological, and neuropsychiatric diseases [10].

Although the topic of enterotypes is still debated within the scientific community, at least 3 ways of classifying the microbiota have been proposed over the past 10 years and will be discussed in this narrative review: a first compositional model with two of three enterotypes with dominance respectively expressed by *Bacteroides*, *Prevotella*, and *Ruminococcus*; a second compositional “open” model and able to visualize two, three, and even four different clusters, according to the different analytical models used; and a third compositional model, in which, according to a different bacterial load observed, the enterotype 1 (dominated by *Bacteroides*) is split into the enterotypes B1 and B2, with the latter showing a bacterial load significantly lower than the one observed in B1.

## 2. Classification of the Complexity of Fecal Microbiota

Although it is possible to investigate the structure of a fecal microbiota through the parameters of biodiversity and taxonomic bacterial composition by main groups (*phyla*) and genera (*taxa*), the intimate consortium structure of which it is composed makes it rather inaccessible to study. Its complexity is, in fact, such that an approach aimed at simplifying it is still necessary. The first step towards its simplification is classification. Classifying the typical consortium structures of the colon fecal microbiota based on compositional models, in fact, would not only facilitate their structural understanding, but would also enhance the diagnostic aspects, at the same time facilitating the implementation of therapeutic hypotheses with important implications for the personalized treatment to be carried out through nutritional, probiotic, prebiotic, and pharmaceutical interventions. Such microbial composition models could be used to stratify populations, such as the molecular under-typing commonly used in breast cancer research, in which, for example, adenocarcinoma subclasses based on gene expression models are clinically relevant [12,13].

## 3. The Classification of the Fecal Microbiota by Enterotypes

**Figure 1:**
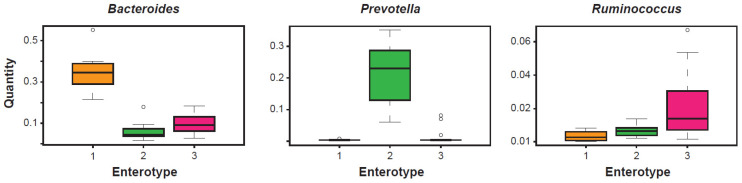
The analysis of gut microbiota of adult and healthy subjects demonstrates the existence of three clusters, or enterotypes, centered on a main driver, respectively *Bacteroides*, *Prevotella*, and *Ruminococcus*, measured for its relative abundance. Modified from [14].

The clustering carried out in 2011 of fecal metagenomic samples from three different continents (Europe, North America, and Asia) were analyzed using three different sequencing technologies (Illumina, 454, and Sanger) and their profiling data were obtained from the 16S rRNA gene. This study led to propose the existence of three apparently well-defined and frequently recurring microbial communities (Figure 1). These clusters were identified as enterotypes [14]. Simply numbered from 1 to 3, these bacterial clusters were independent of the age, gender, cultural background, and geography of their respective hosts. Since then, the concept of enterotype has become part of the typical language of those involved in the analysis and diagnostics of the microbiota. An investigation into the properties of each of these clusters clearly highlights the structural type determined by the existence of covariant bacterial networks centered exactly on the indicator *taxon* (driver), a genus characterizing a given enterotype. In enterotype 1, with *Bacteroides* as the best indicator, covariant bacteria are, for example, *Parabacteroides*, *Alistipes*, and *Bilophila*. In enterotype 2, with *Prevotella* as the best indicator and whose abundance is inversely proportional to *Bacteroides*, covariant bacteria are, instead, *Desulfovibrio* and, sometimes, *Succinivibrio*. In enterotype 3, distinguished from the first two mainly by an over-representation of Firmicutes, the best indicators are *Ruminococcaceae* and *Ruminococcus*, and co-occurring *taxa* are frequently *Akkermansia* and *Methanobrevibacter*. In the complexity of each enterotype, negatively covariant bacteria are also highlighted. *Taxa* of this type are, for example, *Methanobrevibacter* for enterotype 1, *Akkermansia* for enterotype 2, and *Prevotella* for enterotype 3. Enterotypes 1 and 2, both dominated by *taxa* of the *phylum* Bacteroidetes, show abundances in the driver that are sharper than that shown by enterotype 3, in which the dominance of *Ruminococcus* appears less evident. Beyond the different dominance values observed in the three enterotypes, and despite the well-known functional heterogeneity of some genera in the execution of the clustering, the taxon-driver combination was chosen because it is at the *taxon* level that it was hypothesized, and it is still hypothesized, that the microbial ecological niches are reflected more clearly.

Again in 2011, the work of Arumugam, performed on only 39 healthy subjects, was confirmed by a subsequent publication [15]. After having taxonomically profiled the 16S rRNA gene on 139 individuals, the authors arrived at the same conclusions: based on dominance and covariance, the human fecal microbiota tends to form a consortium in 3 possible enterotypes. Among these, enterotypes 1 and 2 (*Bacteroides* and *Prevotella*) appear to be defined, while enterotype 3 (*Ruminococcus*) appears more nuanced and often co-dominant with *Bacteroides*. The presence of *Bacteroides* and *Prevotella* in the corresponding enterotypes highlighted the possible dietary pattern of the hosts, with a certainly more Western diet, therefore rich in animal proteins and fats, in enterotype 1, and a diet more typical of agricultural communities, with a higher content of vegetable polysaccharide fibers, in enterotype 2 [14,15]. This dualism seemed to be justified in the metagenomic results due to which a certain dichotomy between enterotypes 1 and 2 seemed evident, mainly characterized by a greater expression of a genomic machine operating towards fats and proteins, in the *Bacteroides* dominance, and towards polysaccharides and simple sugars, in the *Prevotella* one. This latter evidence seemed to also be confirmed experimentally. Five individuals characterized by enterotype 1 (*Bacteroides*) were in fact subjected to a dietary switch and, for the following ten days, they ate a diet low in animal fats and proteins, and rich in vegetable fibers. In at least 3 out of 5 subjects, the microbiota changed both in richness and in taxonomy starting from the second day, showing a clear trend towards structural change. The enterotype did not shift from 1 to 2 in those 10 days in any individual and the microbiota were overall quite resilient, but the control group (subjects with enterotype 1 and a diet continuously high in animal fats and proteins, and low in fibers) showed no structural variation during the experiment, indirectly demonstrating the strong impact of food typology on the structure of enterotypes. The epidemiological evidence in the *Bacteroides*–*Prevotella* dichotomy is even stronger, as shown by the comparative studies between individuals of agricultural societies and individuals of industrial societies, with the former certainly classifiable as enterotype 2 and the latter more frequently classified as enterotypes 1 and 3 [16].

The two works, respectively performed by Amurugam and Wu [14,15], describing the possible clustering of gut microbiota in bacterial communities more frequently characterized by a main driver and by covariant bacterial elements, have highlighted both the probable presence of three different enterotypes and the perhaps lower consistency, otherwise interpretable as more nuanced, of the Firmicutes (or *Ruminococcaceae* and *Ruminococcus*) dominance with an enterotype 3, which, when merged into enterotype 1, would determine its non-detectability, thus generating a classification of the human microbiota according to a simpler model with only two enterotypes. This double possibility was also highlighted by other authors, who found the human fecal microbiota analytically groupable in three enterotypes (*Bacteroides*, *Prevotella*, and *Ruminococcus*), with a less clear dominance of driver in enterotype 3, and in two enterotypes (*Bacteroides* and *Prevotella*), with almost no evidence of the existence of a third enterotype (*Ruminococcus*) [17,18].

**Figure 2:**
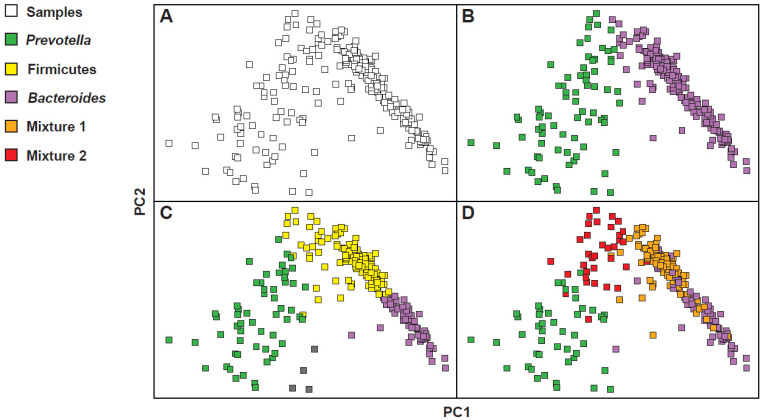
Re-analysis of three different large fecal metagenomic datasets. Although the emergence of community profiles is modest, in certain areas of the graphs obtained by analysing the principal coordinates (PC1 and PC2), certain densities of samples appear. The use of colors makes these densifications more evident. (**A**) gradient-model; (**B**) 2-cluster model; (**C**) 3-cluster model; (**D**) 4-cluster model. Modified from: [19].

However, the clustering of the microbiota in models with two or three enterotypes is not the only possible model. In fact, in other subsequent studies, some authors opted for a clustering according to a model with four enterotypes, while others claimed the existence only of an evident compositional gradient, in the absence of visible clusters [19]. To understand how it was possible to arrive at such different considerations, one should observe the results obtained from the investigations of three large data sets, such as the Human Microbiome Project (HMP), the European Metagenomics of the Human Intestinal Tract project (MetaHIT), and the Chinese Metagenome Wide Association Study (MGWAS) [20,21,22]. As shown in Figure 2, whose configuration was obtained by calculating the distances between the samples, reporting the complex abundance distributions of some bacterial *taxa,* and highlighting the resulting clusters, the gradient is not very clean (Figure 2A) and, differently, at least two obvious masses, one on the right and one on the left, appear quite clearly. Then, resorting to the use of colors, some configurations seem to occur more frequently than others. Some of these coincide exactly with the *Bacteroides*, *Prevotella* and Firmicutes dominant clusters (Figure 2B,C). In the model with four enterotypes (Figure 2D), on the other hand, while the *Bacteroides* and *Prevotella* dominant clusters remain clear and separated, two mixed groupings are configured. The first, called Mixture 1, corresponds to a Firmicutes–*Bacteroides* superposition; the second, called Mixture 2, corresponds instead to an overlap, quantitatively less important than the first, Firmicutes–*Prevotella*. This preference for specific profiles of microbial communities is however modest and a higher density of samples around the preferred constellations also counteracts a certain proportion of samples that fall between them. This makes it difficult to mathematically describe these preferred microbial compositions or to accurately determine the number of such densely populated areas. However, it is important to characterize these pseudo-groupings of bacterial communities to then try to understand their properties and intrinsic ecological constraints. Note, however, precisely in relation to the properties of these clusters, that in the three-cluster model here described there is only a partial correspondence with the classical clustering in which enterotype 3 has a dominance of *Ruminococcus* and Ruminococcaceae. In the three-cluster model used to justify also the four-cluster model, more than a dominance of *Ruminococcus and* Ruminococcaceae, an enlarged dominance of the *phylum* Firmicutes is observed. This *phylum* is variously split together with the other two clusters, giving rise to the four-cluster model. Understanding the different models used is important. In this case, for example, it justifies how, in the original model [14], the enterotype 3 (*Ruminococcus*) is the least frequent in the population, while, in the modelling used to explain the possible “multiple” interpretations of the bacterial landscape [19], the Firmicutes cluster is both the most frequent and the most decisive one in the visualization of the four-cluster model.

**Figure 3:**
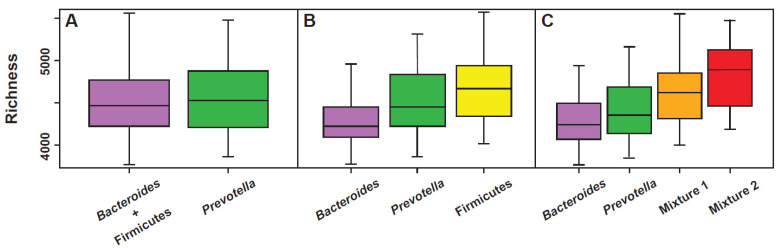
Relationship between functional richness and gut microbiota clusters evaluated according to the models that consider the existence of 2 (**A**), 3 (**B**), or 4 (**C**) clusters. Modified from [19].

All the models used to explain the colonic fecal bacterial landscape through the enterotype, both the original three-enterotype model [14,15] as well as the “multi-cluster model” proposed by Costea [17], can be considered valid, but, in the manner of all the models, they certainly present specific strengths as well as certain weaknesses. For example, a perfect correlation with the richness parameter is evident in the multi-cluster model (Figure 3). The same model, however, is unable to accurately highlight the presence of methanogens, likely markers of a slowed bowel motility [23]. On the contrary, this second aspect is well highlighted in enterotype 3 (*Ruminococcus*) of the original model, in which *Methanobrevibacter* is positively covariant [24]. As previously described, however, depending on how one decides to visualize the compositional elements of the microbiota, the landscape of the enterotypes can also show, at least in part, the characteristic of the “gradient” (Figure 2A) and this option cannot be completely ruled out. The issue of a continuous gradient has been addressed and has led some authors to affirm that “the issue of the enterotype should be completely rethought” [25]. In fact, while admitting that the classification of the human intestinal microbiota into distinct enterotypes could provide an interesting framework for understanding bacterial variation and its correlations with aspects of human health and disease, some methods of mathematical and bioinformatic analysis would instead show the collapse of the microbial landscape in a continuous gradient [25]. However, life sciences show many phenomena, for example the age of individual or the progressive evolution of species, indeed corresponding to a continuous gradient. Despite this, and with scientific advantage, they are anyway clustered.

The number of models proposed to cluster the gut microbiota in a compositional manner is certainly useful, but maybe the three-cluster model, the one considering Firmicutes instead of *Ruminococcus*, allows to derive some important correlations of a translational nature, at least if we consider the aspects of apparent increase in the risk of developing a specific pathology. For example, the *Bacteroides* cluster has been correlated with non-alcoholic steatohepatitis or NASH [26], low-grade inflammation [21], colorectal cancer [27], chronic inflammatory bowel disease [28], celiac disease [29], and immuno-senescence [30]. The *Prevotella* cluster has, instead, been correlated with hypertension [31], rheumatoid arthritis [32], insulin-sensitivity [33], and HIV infections [34], especially when intercepted in male homosexuals [35]. Finally, the Firmicutes cluster has been correlated with a higher risk of incurring atherosclerotic diseases [36].

## 4. Enterotypes: Stability and Phenotype

Except for some rare observations about the existence of an “H” enterotype with dominance expressed by *Enterobacteriaceae* [37], a cluster probably to be considered strongly dysbiotic and linked to incorrect lifestyles (for example, alcoholism) or to the presence of pathologies (for example, obesity and/or NASH) [19], the compositional structures most frequently observed in the human microbiota are dominated by *Bacteroides*, *Prevotella*, and Firmicutes or—and in this sense we speak more properly of enterotypes—dominated by *Bacteroides*, *Prevotella*, and *Ruminococcus*. One of the aspects that is most relevant when discussing microbiota clustering and/or enterotypes is their stability. Unfortunately, an important limitation to most of the studies performed on human enterotypes, however, is the lack of longitudinal data. Much of what has in fact been observed and described is the result of snapshots that cannot describe intrinsic stability aspects. To the extent that it is desired to obtain robust translational information from the enterotype clustering, knowing its stability, or its propensity to veer and therefore to be “fluid”, becomes fundamental.

As far as it is known, the gut microbiota of healthy adults is characterized by some degree of stability with little propensity for fluidity [15,38,39]. Longitudinal evaluations that were conducted for at least 6 months have clearly shown that about 85% of fecal samples do not change in structure and enterotype, and the aspect of fluidity would seem to concern only the remaining 15% [19]. Within this possible propensity to change enterotype, the most probable switches are those involving the Firmicutes dominance to become a *Bacteroides* dominance. The reverse, from *Bacteroides* to Firmicutes, would be configured with a frequency by 50% lower. *Prevotella* would seem to be the most stable cluster, closely followed by *Bacteroides*. Longer studies suggest the same sort of stability [40]. In fact, metagenomic measurements performed on healthy subjects not only show that 60% of the microbiota is identically present 5 years after the first detection, but that this stability, confirming what has been said previously, is greater for the *phylum* Bacteroidetes. Always evaluated at the *phylum* level, stability is then progressively reduced in Actinobacteria, therefore in Firmicutes and certainly more evidently in Proteobacteria, which, being bacteria facilitated in horizontal transmission, constitute the most fluid *phylum*, as it was legitimate to also imagine in consideration of their capacity to determine infectious pathologies. However, the stability of the microbiota is evident when evaluated in healthy subjects and in whom the diet does not change significantly over the years. As a demonstration of the importance of nutrition in the construction of the gut microbiota, the simple “going on a diet” in fact determines a very strong reduction in microbiota stability. In addition to the food driver, what best contrasts the intrinsic resilience of the fecal microbiota of healthy subjects is the environment. While admitting how strongly the “environment” parameter is intertwined with the “food” one, immigrants that transfer to Western territories, such as the transfer to the USA, produces a sudden reduction in the richness and production of short-chain fatty acids, along with a rapid shift from *Prevotella* to *Bacteroides* dominance [41,42].

About the “phenotypic” characteristics of the various enterotypes, at least of those described based on the original model (*Bacteroides*, *Prevotella*, and *Ruminococcus*), it is possible to note a certain correspondence with their main parameters, such as stability and resilience [43]. For example, as previously mentioned, richness, and consequently also functional redundancy, appear to be certainly higher in enterotypes 2 (*Prevotella*) and 3 (*Ruminococcus*) than in enterotype 1 (*Bacteroides*). However, the rate of growth, that is, the ability to proliferate, is certainly greater in the latter. If, on the one hand, this can translate, for enterotype 1, into a greater propensity to be able to correlate with disease states, as it is more vulnerable to perturbations, on the other hand, it also reveals its rapid ability to recover. Similarly, the low proliferative rate observable in enterotype 3 (*Ruminococcus*) suggests a lower sensitivity to antimicrobials, especially towards those that, to act effectively, exploit the aspects of rapid bacterial growth, but also describe a lower capability to re-growth. Furthermore, the same aspects of low proliferation rate explain why enterotype 3 is more evident in subjects who declare a slowed intestinal motility. If we then evaluate the relationship between the ability to derive energy from saccharolysis and proteolysis, two of the most performing metabolic processes in bacteria, it can be observed that enterotype 3 has an enormous propensity, compared to enterotypes 1 and 2, to proteolysis. This characteristic, as it will be described later, allows to explain, at least in part, the existence of a strong correlation between the Firmicutes cluster (within which there are obviously *taxa* attributable to enterotype 3) and cardiovascular diseases on an atheromatous basis [43]. As it will be shown, enterotype 3 is the one that presents, among all, the greatest capacity to generate, from proteolysis, toxic catabolites, such as, for example, the derivatives of cresol and indole.

Concerning the metabolic potential of the different enterotypes, saccharolysis appears well represented in the *Bacteroides* dominant enterotype and slightly less in the *Prevotella* enterotype. The phenomenon is apparently only in contradiction with what has been observed regarding diet (low in fibers: enterotype 1; rich in fibers: enterotype 2), since the *Bacteroides* enterotype is the one that has the most modest richness and functional redundancy. Both, richness and functional redundancy, are much less expressed in the *Bacteroides* enterotype than in the *Prevotella* enterotype (Figure 3). In regard to the lipolytic potential, this appears extremely low in the *Prevotella* enterotype, a phenomenon that probably contributes to explain the low frequency of this cluster in subjects with a typically Western diet, and similarly expressed both in the *Bacteroides* and *Ruminococcus* enterotypes. Even if, except for a pharmacological inhibition of pancreatic lipases, it is objectively difficult for lipids to reach the colon in large quantities, the discriminant able to differentiate which of these two enterotypes is most involved in lipolysis could be the type of intestinal motility of the host.

## 5. Enterotypes in the Animal World

Enterotypes are not clusters observable only in the human gut microbiota, but, on the contrary, are identifiable in communities throughout the animal world. In laboratory mouse (*Mus musculus*), for example, an animal very often used in microbiota studies, two enterotypes are well distinguished. Identified as ET1 and ET2, they are characterized, respectively, by the co-dominances Lachnospiraceae–Ruminococcaceae and Bacteroidaceae–Enterobacteriaceae. Biodiversity is highest in ET1; inflammatory parameters (calprotectin, for example) are instead higher in ET2. The well-known *Bacteroides*–*Prevotella* antagonism is also present in mice. *Prevotella* is, in fact, present in ET1, being ET2 characterized, on the contrary, by a strong presence of *Bacteroides* [44]. Two enterotypes very similar to those observed in laboratory animals are observed in wild mice, with the *taxa Robinsoniella* (*Lachnospiraceae*) and *Bacteroides* dominating [45].

In the common chimpanzee (*Pan troglodytes*), which, together with the bonobo (*Pan paniscus*), is the closest relative of the genus *Homo*, three enterotypes are clearly highlighted that apparently recall those identified in human beings [46]. In fact, by “centering” the characterization on the *taxa* that best describes, according to the original model [14,15], the enterotypes of humans, that is *Bacteroides*, *Prevotella*, and *Ruminococcus*, some similarities can be observed between “them” and “us”. Anyway, a more careful examination of the complete profiles of these clusters allows to highlight a minimum common denominator abundantly present in the three clusters of the chimpanzee: *Prevotella*. On the contrary, this feature is absent in modern man.

## 6. Possible Evolutionary Aspects of Fecal Microbiota

**Figure 4:**
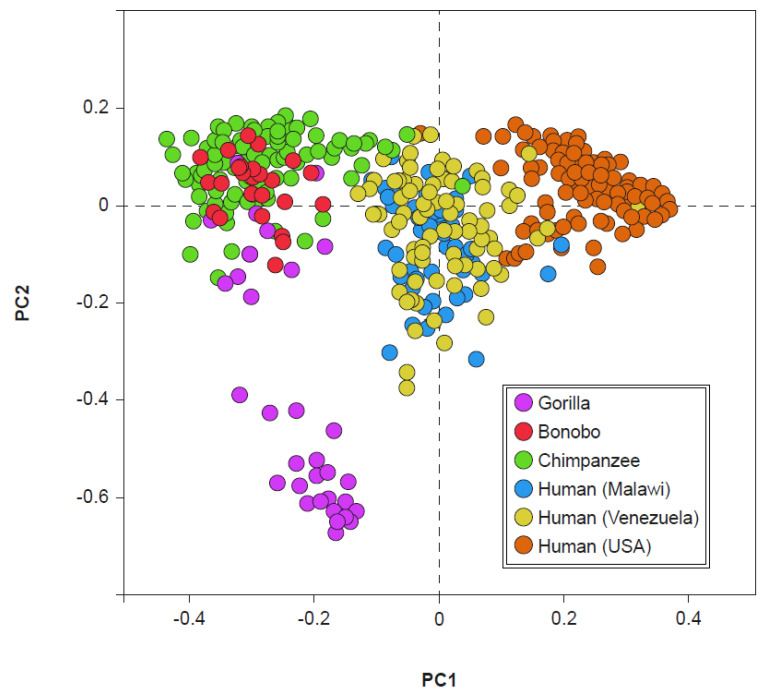
Visualization, through principal coordinates (PC1 and PC2), of gut bacterial consortia of some primates (including humans). Modified from: [47].

The presence of *Prevotella*, which is present in the fecal clusters of the chimpanzee and dominant only in human enterotype 2 (currently very frequently expressed only in the gut microbiota of rural societies), suggests a possible evolutionary link. Indeed, the fecal microbiota of chimpanzees and bonobos show a particularly evident degree of similarity with the fecal microbiota of non-Westernized humans (Figure 4). However, this evidence does not seem to exist between the gorilla and man and between *Pan* (chimpanzee and bonobo) and Westernized human beings [47]. If we consider the divergence in the evolution of microbiota, occurred between 19 and 8 million years ago, which separated the gorillas from the *Pan*/*Homo* group, and the subsequent one, which occurred between 13 and 7 million years ago, which separated the microbiota of *Pan* from those of *Homo*, the lack of an overlap between the microbiota of the gorilla and those of non-Westernized humans can be understood. If, moreover, we take into consideration the current presence of the different clades, simply named A, B, C, and D, of *Prevotella copri* in human microbiota, we observe how these are abundantly represented in the microbiota of modern man only if not Westernized, and in the microbiota of ancient man, as for example in Ötzi (also known as Iceman), datable to approximately 3100 BC, or in some Mexican coprolites dating back to around AD 1300 [48]. This could explain the similarity between the microbiota of the genus *Pan* and those of non-Westernized man, also suggesting a possible evolutionary link of a taxonomic type in which the ancient microbiota, inherited from and currently shared with, the *Pan* genus, could be that of *Prevotella* dominance, which is the current enterotype 2. Similarly, also the gut microbiota richness seems to have evolved directly from the world of primates, in whom it is very high. Furthermore, the richness that is evident in the man of rural societies is slightly lower. Even more reduced is the one that is, finally, evident in the modern Westernized man [47]. A possible explanation for the evident progressive reduction in the biodiversity observed in present-day man, typical of rural societies who does not live in an environment rich in antibiotics or even uses them (and that could evidently have already occurred in ancient man), is the carcass-eating hominid hypothesis [49]. From the marks observed on the teeth of the first hominids, the hypothesis of individuals beginning to taste the flesh of dead animals seems plausible. One thing that modern man has in common with carcass-eating animals, such as hyenas and vultures, is an extremely low gastric pH, close to 1 [50]. The strong gastric acidity protects the carcass eaters, and therefore also the first hominids, from the pathogens potentially present in rotting meat, mainly Proteobacteria. Yet, it probably also contributes to a reduction in fecal bacterial diversity. Like richness, the bacterial load (likely correlated in man with displeasing aspects, such as meteorism and flatulence, and with painful phenomena, such as abdominal distension) also tends to decrease as we proceed from primates towards man [50]. This change appears to have occurred in the dependence on the modification in intestinal length. In primates, in fact, the ratio between small and large intestine is between 1:1 or 1:2. Differently, in humans this ratio becomes approximately 7-8:1 due to an enormous development of the small intestine and the corresponding shortening of the colon. The process would have been triggered by the cooking of food [51]. Cooking makes food easier to assimilate in the small intestine area, a phenomenon that reduces the importance of the colon for developmental purposes. Evidently, colon size reduction consequently produces the decrease in the bacterial load.

**Figure 5:**
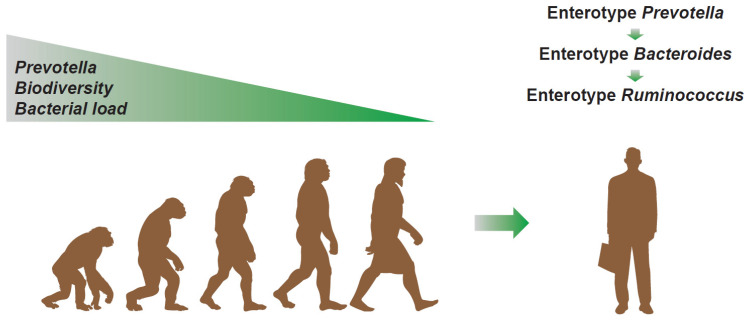
Evolutionary hypothesis of the human gut microbiota based on the parameters of dominance, biodiversity, and bacterial load and based on the structure of the enterotype.

Therefore, from an evolutionary perspective (see Figure 5), it is plausible to hypothesize that the microbiota perhaps starts from a *Prevotella* dominant condition, with high biodiversity and bacterial load, and, from there, it progressively evolves, losing these characteristics. In fact, during the evolutionary process, man begins to eat the meat of carcasses and develops a very low gastric pH, a phenomenon that makes the first contribution to the reduction in the biodiversity of bacteria. The cooking of food, which is responsible for a large part of the telencephalic development, then determines the shortening of the colon to the benefit of the small intestine. This transition determines the strong reduction in the total bacterial load. In small steps, this evolution also proceeds in modern times, in which, due to an increasingly evident reduction in dietary fibers, a transition from enterotype 2 to enterotype 1 is observed [52]. The antibiotic era, capable of selecting bacterial patterns with slower proliferation, then perhaps gave way to a new transition from enterotype 1 to enterotype 3. The latter, numerically less evident, still retains the “scar” of *Bacteroides* often co-dominant with *Ruminococcus* and *Ruminococcaceae*. On the contrary, the absence of *Prevotella*, rarely co-dominant in enterotype 3, indicates the possible correctness of this view. Enterotype 3, however, shows a recovery of richness to levels more frequently observed in enterotype 2 (*Prevotella*), typical, instead, of the man who lives in rural areas and eats a diet rich in dietary fibers. This aspect could be linked, as previously mentioned, to a selection process perhaps connected to the environment increasingly saturated with antibiotics, but also, more likely, to an increasingly prevalent condition of constipation, probably due to an increasingly stressful lifestyle and to an increasingly clear lack of fibers in the diet.

## 7. Correlation between Enterotypes and Fecal Consistency

One of the aspects that most correlates with enterotypes is fecal consistency [53]. This is commonly measured through the Bristol Stool Score (BSS), a diagnostic tool created for the purpose of classifying the shape and consistency of human stools according to seven distinct categories [54]. In simple terms, in type 1, the stool appears as hard and separated lumps, in the shape of nuts or hazelnuts that are difficult to expel; in type 2, the lumps are joined together and the difficulty of expulsion, less than in type 1, still partly persists; in type 3, they are salami-shaped with possible cracks on the surface, while the difficulty of expulsion is absent; in type 4, the stool are sausage- or snake-shaped, smooth and soft, and are easily expelled leaving the sensation of complete intestinal emptying; in type 5, they are fragmented and the fragments are soft and separated from each other and with well-defined margins, and evacuation is very easy; in type 6, the stools are pasty and jagged with floccular or shapeless pieces, the edges are mostly irregular, and the evacuation is diarrheal; in type 7, the fecal material is liquid, with no solid parts, and evacuation is defined as dysenteric. If the BSS is correlated with richness, it can be observed that this is higher in type 1 and progressively decreases as one proceeds towards BSS type 7. The BSS–enterotype correlation shows that, in BSS 1 and 2 (constipation), the presence of the *Prevotella* or *Bacteroides* dominance is generally not found, or rarely found. More frequently, the BSSs representative of constipation correlate with the *Ruminococcus* enterotype. Regardless of the enterotype, the correlation between the BSS and *phyla* or *taxa* correlates perfectly and does not contradict what has just been observed. For example, as the Firmicutes/Bacteroidetes ratio increases, the BSS value decreases, and the same is evident as the value of *Ruminococcaceae* increases. The greater the relative presence of *Bacteroides*, the greater the BSS value; on the contrary, the greater the presence of *Akkermansia*, positively co-occurring in enterotype 3 and a negative covariant with enterotype 2, the lower the BSS value. The greater the relative presence of *Methanobrevibacter*, a negative covariant in enterotype 1 and positive in enterotype 3, the lower the BSS [53].

If we evaluate the transit times of the general population, using telemetry capsules capable of detecting pH and oxygen tension, we observe that about 75% of the population evacuates within the first 24 h after ingestion; 15% evacuates over the next 24 h; and the remaining 10% evacuates in progressively longer times and, in any case, rarely more than 96 h after ingestion [52]. If we parameterize the times measured in the gastric, duodenal, and colonial environments, regardless of the results obtained on the time required for evacuation, the colon transit, almost exclusively, makes the difference [55]. This is the reason for the strong correlation between biodiversity, enterotype, and the BSS. The enterotype is, in fact, an analysis of the colonic material and the BSS is an evaluation that reflects the transit speed, which is mainly influenced by colonic times [56].

## 8. The Correlation between Enterotype 3 and Cardiovascular Risk

If the results of metabolomic studies carried out in subjects with constipation are observed, it is easy to highlight the strong presence of metabolites from protein catabolism, such as p-cresol-sulphate, p-cresol-glucuronide, indole-3-carboxylate-glucronide, 6-hydroxy -5-methoxindole-glucuronide, phenylacetylglutamine, 5-methoxindolacetate, N-phenylacetyl-glutamate, and dimethyl-sulphone [56]. These compounds are derived from the putrefactive metabolism of the colonic microbiota, capable of metabolizing proteins of food and/or endogenous-tissue origin, present in the colon environment [57]. Another evident feature in these subjects is the strong reduction in the presence of metabolites deriving from the catabolism of mucin glycans, such as N-acetyl-galactosamine-sulphate, sialyl-N-acetyl-lactosamine, sialyl-lactose, and N-Acetylneuraminic acid [56]. In subjects with constipation, therefore, protein catabolism increases with the release of compounds, derivatives of sulphur and nitrogen, considered toxic to the host [58] and the metabolic availability of mucin glycans seems to be depleted. The first phenomenon is linked to the fact that bacteria prefer to primarily metabolise carbohydrates and opt for a “lunch” based on proteins only when these are finished. Carbohydrates (essentially polysaccharide fibers) terminate, even regardless of the quantity ingested, in strict dependence on the colonic transit times. In constipation, especially if severe, the fibers always run out and this determines the onset of protein catabolism. In constipation, bacteria with a slow replication rate and those co-occurring in enterotype 3 are mainly selected (*Ruminococcaceae*, *Christensenellaceae*, *Clostridiales* and *Methanobrevibacter*). Indeed, exactly these microorganisms correlate perfectly with the release of these catabolites [56]. The second phenomenon is likely due to the quantitative reduction in mucin, with the probable thinning of the supra-enterocytic mucus layer. The mucus, whose function is to keep the “host” compartment separate from the potentially infecting “bacterial” one and to reduce the risk of poisonous compounds meeting the host’s cells, could be reduced due to two possible, but different, processes. In the first, the slowed motility of the fecal mass could “remove” less mucus, also resulting in a lower mucosal cell turnover. A smaller amount of mucus would then become available to be metabolized. The lower mechanical removal of mucus, by means of non-feedback mechanisms, would progressively reduce the action of the Goblet cells, whose function is precisely to synthesize de novo mucus. The observation that rats treated with loperamide, a known intestinal motility slower, produce less mucus than normal would support this first assumption [59]. In the second, perhaps more likely, the mucus becomes thin for opposite reasons. The absence of polysaccharide fibers, exhausted by the long stay of the fecal mass in the colon, selects some mucus-eating bacteria (mucin glycans are chemically similar to polysaccharide fibers and the bacterial greed for polysaccharides drives them, in the absence of fibers, to feed on mucus), whose catabolic action could be greater than the anabolic capacity of the Goblet cells with the consequent progressive reduction in the mucus layer. The observation that in rats the high-protein and fiber-free diet thins the mucus layer would support this second hypothesis [60]. The two mechanisms could evidently also be concomitant or appear alternately. Hypotheses aside, the absence of fiber physically produces a reduction in the mucus layer [61]. In conclusion, a slowed intestinal motility contributes to select a microbiota characterized by a high biodiversity and by a clustering described as enterotype 3. In this microbial landscape, we observe the exhaustion of the polysaccharide fiber, a pronounced protein catabolism that generates compounds toxic to the host, and the thinning of the protective mucus layer. These consequences, of course, significantly affect the morbidity and mortality of subjects with other cardiovascular risk factors, such as hemodialysis patients [62]. The analysis of the microbiota, therefore, performed on subjects with previous cardiovascular risk factors would perhaps allow to intercept those subjects with additional risk factors correlated with a specific microbial consortium, with high richness and with a pronounced enterotype 3. The possible plausibility of what is here stated is easily confirmed by the most extensive epidemiological survey ever conducted in this area (performed on 3.4 million subjects) and aimed at correlating slowed intestinal motility with the incidence of serious cardiovascular events. The results show that, in subjects with slowed colonic transit, the incidence of potentially fatal vascular events is increased by 10–20% depending on the event considered, with a 12% increased mortality compared to controls [63].

## 9. Enterotype B2 and Its Possible Correlation with the Anxiety-Depressive Syndrome

Higher biodiversity, a parameter conventionally considered positive, cannot be considered “by definition” and, on the contrary, in certain conditions, in which it would appear to be only a “deformed” parameter by slowed colon motility, it could constitute a possible alarm signal. In addition to the various drivers capable of influencing the biodiversity of the stool consortium (variety in the diet, ingestion of fiber, use of antibiotics, and colon motility), diversity is also characterized by elements that show a proportional trend with it. The best known of those is the bacterial load. In fact, as described before, as the richness increases, the bacterial load also grows. A reverse trend with respect to biodiversity is instead shown by stool hydration. In fact, a 20% reduction in hydration corresponds to an approximately three-fold increase in the bacterial load per gram of stool [64]. Therefore, if the originally described enterotypes (*Bacteroides*, *Prevotella*, and *Ruminococcus*) are evaluated based on an absolute quantitative analysis of the bacterial load, two different *Bacteroides* enterotypes appear. Named respectively B1 and B2, they are differentiable on the basis to the absolute bacterial load, with higher values in the first and reduced values, about tenfold, in the second [64]. According to this model, the enterotypes dominated respectively by *Prevotella* and *Ruminococcus* are indicated by the authors simply as P and R. Simply put, the B2 enterotype is characterized by a *Bacteroides* dominance and soggy stools, therefore high hydration and low bacterial load. Taxonomic parameters describing the B2 enterotype are a reduction in Firmicutes, especially *Faecalibacterium*, *Dialister*, and *Coprococcus*. This further modality of clustering the microbial landscape is potentially capable of intercepting subjects with poor quality of life and at risk of anxious-depressive syndrome. In fact, among depressed subjects, the B2 enterotype demonstrates a double incidence compared to that seen in healthy subjects. On the contrary, the enterotype B1, and those dominated by *Prevotella* and *Ruminococcus*, show a lower incidence in depressed people than in healthy subjects [65]. A very recent meta-analysis has confirmed this perspective [1].

## 10. The *Bacteroides*–*Prevotella* Antagonism

The simplest compositional model to which the possible microbiota clusters can be traced is, however, certainly the one with two enterotypes, with the main driver centered on *Bacteroides* and *Prevotella*. These two bacterial *taxa* are often described as antagonists and, above all, respectively pushed to proliferate in gut microbial consortia based on the host’s food style, the first western and the second rural [66]. Unfortunately, this is certainly an oversimplification of the reality. What they certainly differ in is the heterogeneity of the easily colonizable environments, with *Prevotella* potentially dominant also in the oral (*Prevotella oralis*) and vaginal (*Prevotella bivia*) as well as stool (*Prevotella copri* and *Prevotella stercorea*) consortia, unlike *Bacteroides*, a *taxon* more exclusively faecal, which can be detected in the oral and vaginal consortia only in traces. However, if we investigate the *Prevotella* and *Bacteroides* clades, both isolated from the stool consortia of healthy subjects who declare themselves vegan or omnivorous, we certainly observe a constant correspondence of “vegan-*Prevotella*” and “omnivore-*Bacteroides*”, but also, and not only rarely, *Prevotella* clades well characterized in omnivorous subjects and well characterized *Bacteroides* clades in vegan subjects [67]. The analysis of these clades demonstrates a strong genomic correspondence with food. The *Prevotella* clades found to be abundant in omnivores demonstrate a pronounced presence of genes active in the metabolism of proteins and fats, while the clades of *Bacteroides* found abundantly in vegans demonstrate a strong gene expression in the metabolism of fibers. The expression of genes to produce trimethylamine also correlates in the same way: it is strong in *Prevotella*, which is abundantly present in omnivores, and weak in *Bacteroides*, found abundantly in vegans. The various clades are also potentially traceable, obviously in different percentages, in both types of subjects, vegans and omnivores. Based on their presence, they co-occur each other, positively or negatively. Therefore, regardless of being rich in *Prevotella* or *Bacteroides*, *Prevotella* clades with “omnivorous” genes co-occur positively with each other and negatively with *Prevotella* clades endowed with “vegan” genes. Of course, the same is valid for the *Bacteroides* clades [65]. This clearly demonstrates how the host’s eating style can only partially explain the detection of a *Bacteroides* enterotype rather than *Prevotella*. It also demonstrates that other environmental elements are probably equally involved. Bile, for example, is likely a factor capable of selecting *Bacteroides* with respect to *Prevotella*. Indeed, *P. copri* and *P. stercorea* show growth difficulty on agar with bile, unlike *Bacteroides* strains [68]. As it is well known, the release of bile is triggered by a meal rich in fats and proteins and this would explain, at least in part, the greater frequency of isolation of the genus *Bacteroides* in subjects with a Western diet compared to those with a rural diet [69]. The presence, or the absence, of bicarbonate (HCO_3_^−^) is certainly another factor capable of selecting between *Bacteroides* and *Prevotella*. Indeed, *Prevotella* does not grow in vitro in the absence of bicarbonate, unlike *Bacteroides* for which, however, it does not seem to be essential [70]. This different dependence could be linked to the different metabolic capacities. The genus *Bacteroides* primarily produces propionate. This is the final product of its ability to metabolize sugars. It obtains propionate from succinate and this metabolic step releases CO_2_. The metabolism and production of short-chain fatty acids, therefore, makes *Bacteroides* self-sufficient in terms of bicarbonate. Differently, some *Prevotella* species stop its ability to metabolise sugars to succinate and is, therefore, more dependent on the bicarbonate present in the environment. The mean stool bicarbonate concentration in healthy subjects varies between 10 and 40 mM, but *Prevotella* growth appears impaired below 20 mM [70]. The members of the Bacteroidetes *phylum*, therefore both *Bacteroides* and *Prevotella*, bear the low pH less well than, for example, the members of the Firmicutes *phylum* do [71]. The more frequent detection in Western subjects of *Bacteroides* compared to *Prevotella* could therefore also be linked to this aspect of greater or lesser dependence on the environmental presence of bicarbonate. Stress, large meals, and a high-protein diet probably predispose an individual to a particularly low gastric pH and the bicarbonate present in the intestine plays a possible buffer role. The self-sufficiency of *Bacteroides* with respect to the possible presence of environmental bicarbonate contributes to favoring it in the microbial consortia found in subjects on a Western diet. Conversely, a less aggressive gastric pH requires a less consistent buffering effect of the environmental bicarbonate. This can feed the metabolic needs of *Prevotella* more, which is therefore more frequently found in the microbiota of non-Westernized subjects. In human tissues, including the intestine, there is a pump that exchanges chlorine for bicarbonate. The gene encoding for this channel is altered in cystic fibrosis [72] and, for this reason, it is called CFTR (cystic fibrosis transmembrane conductance regulator). Therefore, in subjects with cystic fibrosis, a lower release of bicarbonate occurs in the gut. This element should, at least theoretically, put *Prevotella* at a disadvantage. Indeed, the analysis of the microbiota of subjects with cystic fibrosis reveals, compared to controls, the almost absence of *Prevotella* and a high *Bacteroides* content [73,74].

## 11. Conclusions

In recent years, our ability to understand the structure of the human gut microbiota has grown dramatically. The various phases of the Human Microbiome Project, and what has followed, have produced such results that they could soon be translated into clinical practice and public health programs [75]. While it remains essential to be able to distinguish reality from hypotheses (and, in some cases, from excesses and hyperboles), it is necessary to be able to identify the existence of parameters on which to approach therapeutic choices. Based on the various models available, enterotypes could in some cases provide some important indications (Table 1). For example, the enterotype 1 of the original models proposed by Arumugam [14] and by Wu [15] seem to correlate with an increased risk of NASH [26], metabolic endotoxemia [21], and several gut diseases [27,28,29]. The enterotype 2 seems to correlate with hypertension, rheumatoid arthritis, and insulin sensitivity [31,32,33]. Lastly, the enterotype 3 seems to correlate with a greater resilience towards antibiotic therapy, but also highlights a richness influenced by slow intestinal motility, a condition that determines a greater predisposition for the host towards cardiovascular diseases [35]. This last aspect has a clear correlation also with the cluster dominated by Firmicutes according to the multi-cluster model proposed by Costea [19]. Similarly, the enterotype B2, obtained by evaluating the concept of enterotype 1 (*Bacteroides* dominance) with information on stool hydration and therefore on the bacterial load, would predispose the host towards the anxious-depressive syndrome and/or a low quality of life [65]. Although there is still an important scientific debate to be conducted on the concept of enterotypes, these, integrated in their various models, could become a tool to better understand the existence of predispositions in individuals towards certain pathologies.

## Figures and Tables

**Table 1:** Compositional models, bacterial dominance, diet, and possible disease link.

Compositional Model	Dominance	Correlation with
*Enterotypes 1, 2 and 3* [14]	*Bacteroides*	Western diet [14]
	*Prevotella*	Agrarian societies [14]
	*Ruminococcus*	
*Enterotypes 1 and 2* [15]	*Bacteroides*	Western diet [15]
	*Prevotella*	Agrarian societies [15]
*Multi-clusters model* [19]	*Bacteroides* and *Prevotella*	
	*Bacteroides*	ME [21], CRC, IBD, CD [26,27,28,29]
	*Prevotella*	HyT [31], RA [32], HIV [34]
	*Firmicutes*	Constipation [23], CVD [36]
	*Bacteroides*, *Prevotella*, M1, M2	
*Enterotypes B1, B2, P, R* [64]	*Bacteroides* (hbl)	
	*Bacteroides* (lbl)	Depression [65]
	*Prevotella*	
	*Ruminococcus*	

Abbreviations used: ME: metabolic endotoxemia; NASH: non-alcoholic steatohepatitis; CRC: colon-rectal carcinoma; IBD: inflammatory bowell disease; CD: celiac disease; HyT: Hypertension; RA: reumatoid arthritis; CVD: cardiovascular diseases; M1: Mixture 1; M2: Mixture 2; hbl: high bacterial load; lbl: low bacterial load.
